# Japan Diabetes Outcome Intervention Trial-1(J-DOIT1), a nationwide cluster randomized trial of type 2 diabetes prevention by telephone-delivered lifestyle support for high-risk subjects detected at health checkups: rationale, design, and recruitment

**DOI:** 10.1186/1471-2458-13-81

**Published:** 2013-01-29

**Authors:** Naoki Sakane, Kazuhiko Kotani, Kaoru Takahashi, Yoshiko Sano, Kokoro Tsuzaki, Kentaro Okazaki, Juichi Sato, Sadao Suzuki, Satoshi Morita, Kazuo Izumi, Masayuki Kato, Naoki Ishizuka, Mitsuhiko Noda, Hideshi Kuzuya

**Affiliations:** 1 Division of Preventive Medicine, Clinical Research Institute, National Hospital Organization Kyoto Medical Center, Kyoto, Japan; 2 Hyogo Health Service Association, Hyogo, Japan; 3 Department of General Medicine/Family & Community Medicine, Nagoya University Graduate School of Medicine, Nagoya, Japan; 4 Department of Public Health, Nagoya City University Graduate School of Medical Sciences, Nagoya, Japan; 5 Department of Biostatistics and Epidemiology, Yokohama City University, Yokohama, Japan; 6 Office of Strategic Outcomes Research Program, Japan Foundation for the Promotion of International Medical Research Corporation, Tokyo, Japan; 7 Department of Diabetes and Metabolic Medicine, National Center for Global Health and Medicine, Tokyo, Japan; 8 Biostatistics, Biostatistics & Programming Clinical Sciences & Operation Research & Development, Sanofi K.K, Tokyo, Japan; 9 Diabetes Research Center, National Center for Global Health and Medicine, Tokyo, Japan; 10 Koseikai Takeda Hospital, Kyoto, Japan

## Abstract

**Background:**

Lifestyle modifications are considered the most effective means of delaying or preventing the development of type 2 diabetes (T2DM). To contain the growing population of T2DM, it is critical to clarify effective and efficient settings for intervention and modalities for intervention delivery with a wide population reach.

The Japan Diabetes Outcome Intervention Trial-1 (J-DOIT1) is a cluster randomized controlled trial to test whether goal-focused lifestyle coaching delivered by telephone can prevent the development of T2DM in high-risk individuals in a real-world setting. This paper describes the study design and recruitment of the study subjects.

**Methods:**

For the recruitment of study subjects and their follow-up annually over 3 years, we employed health checkups conducted annually at communities and worksites. Health care divisions recruited from communities and companies across Japan formed groups as a cluster randomization unit. Candidates for the study, aged 20-65 years with fasting plasma glucose (FPG) of 5.6-6.9 mmol/l, were recruited from each group using health checkups results in 2006. Goal-focused lifestyle support is delivered by healthcare providers via telephone over a one-year period. Study subjects will be followed-up for three years by annual health checkups. Primary outcome is the development of diabetes defined as FPG≥7.0 mmol/l on annual health checkup or based on self-report, which is confirmed by referring to medical cards.

**Results:**

Forty-three groups (clusters), formed from 17 health care divisions, were randomly assigned to an intervention arm (22 groups) or control arm (21 clusters) between March 2007 and February 2008. A total of 2840 participants, 1336 from the intervention and 1504 from the control arm, were recruited. Consent rate was about 20%, with no difference between the intervention and control arms. There were no differences in cluster size and characteristics of cluster between the groups. There were no differences in individual characteristics between the study arms.

**Conclusion:**

We have launched J-DOIT1, a nation-wide trial to prevent the development of T2DM in high-risk individuals using telephone-delivered intervention. This trial is expected to contribute to evidence-based real-world preventive practices.

**Trial registration:**

UMIN000000662.

## Background

Type 2 diabetes mellitus (T2DM) is rapidly becoming one of the major health issues of the 21^st^ century [1,2]. A recent survey performed by the Ministry of Health, Labour and Welfare has projected that approximately 8.9 million people have diabetes and another 13.2 million people are at high risk for diabetes in Japan [3,4]. There is an urgent need for effective strategies to combat this pandemic. The Finnish Diabetes Prevention Study (DPS) [5] and US Diabetes Prevention Program (DPP) [6] both clearly showed that intensive lifestyle intervention can prevent or delay the development of T2DM in a high-risk population. Thus, lifestyle modifications are considered the most effective means of delaying or preventing the development of T2DM [7,8]. The DPP and DPS interventions have been translated into church [9], weight loss clinic [10], YMCA [11], primary care [12], and community [13] settings. However, translating the findings of clinical research, such as the DPS and DPP, into a real-world practice [14] on a large-scale still remains to be addressed. Japan has adopted the universal medical care insurance system, where all the people are insured by one of the public medical insurance systems [15,16]. In 2003 the Health Promotion Law was enforced aiming at preventing lifestyle-related diseases including T2DM. Now it has become mandatory for all Japanese adults to undergo health checkups provided by public medical care insurance at least once a year. There are two main types of statutory health checkup programs; 1) workplace health checkup programs managed by employers (companies setting), and 2) community health checkup programs managed by municipalities (communities setting) for self-employed, unemployed and retired individuals. People are registered at health care divisions in their workplaces or communities, and through the health care divisions, health checkups are provided. Health checkups are becoming part of routine health care. As a whole about 50% of adults undergo health check-ups annually. A large number of high-risk subjects for diabetes are identified every year through these health checkups. It is questionable, however, to what extent annual health checkups contribute toward overcoming the pandemic of diabetes. There is a big gap between identifying high risk subjects and preventing diabetes in the real world. One of the reasons for this may be a lack of evidence-based effective and efficient prevention programs which are easily accessible. The Japan Diabetes Outcome intervention Trial-1 (J-DOIT1) is a nation-wide, cluster randomized controlled trial [17], aiming to establish effective and efficient programs to prevent the development of T2DM in high-risk individuals through lifestyle modifications. The cluster randomization design has the advantages of administrative convenience, ease of obtaining the cooperation of investigators, enhancement of subject compliance, and avoidance of treatment contamination [18]. Health care divisions recruited from communities and companies across the country formed groups as a cluster randomization unit. The data of annual health checkups obtained from each group are utilized for identifying high-risk individuals and follow-up. This paper presents the study protocol in detail, including the rationale and the recruitment results. As a national project, this information should be widely referred to and shared by researchers and practitioners in preventive medicine.

## Methods

This study has been approved by the Ethical Committee of the Japan Foundation for the Promotion of International Medical Research Cooperation (National Center for Global Health and Medicine, Tokyo, Japan).

### Study design

The present study is a cluster randomized controlled trial [19,20] aimed at involving Japanese men and women, aged 20-65 years, at high risk for developing T2DM. For the recruitment of study subjects and their follow-up, we employ health checkups conducted annually by health care divisions at communities and worksites. A total of 17 health care divisions were enrolled across the country. Large health care divisions, with a large number of examinees and branches covering different areas, were divided into groups. A total of 43 groups were thus formed from 17 health care divisions, with each group having approximately 1,000-6,000 annual health checkup examinees. For the cluster randomization, these groups were randomly allocated to either an intervention or a control arm. Using the 2006 health checkup data obtained from each cluster, lifestyle support centers sent a program kit to the candidates who met the eligibility criteria and invited them to participate in the study. The kit included an explanation about the study’s aims and protocol, a consent form, and a questionnaire regarding lifestyle and health status. Those who consented to participate and completed the questionnaire were registered as study participants at lifestyle support centers, after their eligibility was checked based on their self-reported health status. Subjects in the intervention arm will receive non-face-to-face intervention via telephone or mobile-phone over the course of one year. Subjects in control arm will receive no such intervention. The progression to diabetes will be monitored by an annual health checkup and questionnaire over three years. All data for the study are collected at the lifestyle support centers and sent to the data management center in a de-identified form.

### Recruitment of health care divisions

By advertising on the internet or by direct contact, we invited health care divisions at communities and companies to participate in the study. The inclusion criteria for the participating health care division were; 1) it conducts health checkups according to guidelines by the Health Promotion Law, 2) as a rule it has 2,000 or more examinees annually, 3) it can provide the study group with health checkups data every year starting from 2006, and 4) it can conduct lifestyle survey every year using a questionnaire prepared by the study team. Health care divisions, in which study team members are directly involved as industrial physicians, were excluded. Seventeen health care divisions, widely distributed throughout the country, agreed to participate in the study. Among them 14 health care divisions belonged to companies, 2 to municipalities, and 1 was a mixture of small-sized companies and municipalities. They were all approved by the steering committee. A large health care division, covering many distant areas, was divided into groups. This process was done by the health care division itself mainly based on the area and number of examinees. A total of 43 groups were thus formed from 17 health care divisions. The number of groups formed in each health care division ranged from 1 to 10. Each group included 700 to 6,000 annual examinees. Some groups that were small were pooled with others. Using the results of health checkups in 2006, candidates who met inclusion criteria (described later) were identified in each group.

### Randomization

For cluster randomization, the groups were randomly allocated to either an intervention (n=22) or a control (n=21) arm. Randomization was performed 3 times according to 3 recruitment periods (March to April, May to June, and July to August in 2007). When two or more groups were made from one health care division, they were allocated to each of the arms within the health care division. Some small groups were pooled with others. Allocation was carried out using stratified randomization with seven strata of companies or communities in the first period, five strata in the second period, and three strata in the third period. A randomization list was prepared by an independent statistician using the SAS PLAN procedure with seed = 4989. This procedure was conducted using SAS version 9.1 (SAS Inc., Cary, NC, USA). Simple randomization was performed with 2 levels of treatment. The groups were notified of their allocation status before study subjects were recruited. The subjects were notified of their allocation status when they were recruited.

### Health checkups

Guidelines for health check implementation were announced in 2004 based on the Health Promotion Law. In 2006 mandatory items to be checked included 1) anamnesis of past history including history of medication and smoking, 2) subjective and objective symptoms, 3) body height and weight, 4) Body Mass Index (BMI), calculated as body weight (kg) divided by square of body height (m^2^), 5) blood pressure, 6) serum alanine aminotransferase, asparate aminotransferase and gamma glutamyltranspeptidase, 7) serum triglycerides, HDL cholesterol and LDL cholesterol, 8) fasting plasma glucose, and 9) urinalysis. At health checkup sites anthropometric measurements were done by public health nurses or industrial nurses. Height was measured in the standing position by public health nurses or industrial nurses. Weight was measured without shoes or heavy clothes to the nearest 0.1 kg using standard calibrated scales. Systolic and diastolic blood pressure values were measured in the sitting position [21]. Blood was withdrawn after 8 hours of fasting and analyzed with standard methods in clinical laboratories under the nationally certified laboratory management system. If blood was withdrawn from people who had not fasted, plasma glucose data was treated as casual plasma glucose and triglycerides values were omitted from the analysis. We did not perform any additional tests for this study.

### Inclusion and exclusion criteria for study subjects

Using the 2006 year health checkups data, candidates who met the inclusion criteria were identified in each cluster. Inclusion criteria included an age of 20-65 years and impaired fasting glucose (IFG) defined as a fasting plasma glucose concentration (FPG) of 100-125 mg/dL (5.6-6.9 mmol/L). In the 2006 year health checkups, however, blood sampling was not always done in the fasting state. In those individuals where the FPG was not available, plasma glucose concentrations (casual plasma glucose, CPG) of 118-143 mg/dL (6.6-7.9 mmol/L) [22,23] were considered eligible. A CPG ≥11.1 mmol/l (200 mg/dl) indicates diabetic type of glucose tolerance according to the report of the committee on the classification and diagnostic criteria of diabetes mellitus [24,25]. A CPG is also used as the risk assessment for cardiovascular disease in Japan [26]. Exclusion criteria included diagnosed diabetes, a previous history of diabetes taking anti-diabetic agents, a HbA1c of ≥ 6.5% [27]. Women with a history of gestational diabetes could be enrolled. Physical or medical conditions that do not allow exercise, pregnancy or possible pregnancy, evidence for of type 1 diabetes mellitus, liver cirrhosis or chronic viral hepatitis (type B or type C), and use of a cardiac pacemaker were also included as exclusion criteria. We also excluded those who had already participated in other lifestyle modification programs and those who could not obtain the approval from their doctors.

### Enrollment of the study subject

We outsourced some parts of the study works to three existing private companies (Tokio Marine & Nichido Medical Service Co., Ltd., National Education Association, INC. VISIT HEALTH Co., Ltd., and Meiji Yasuda System Technology Co., Ltd., Japan). They were all practicing healthcare services. They participate in this study as a lifestyle support center, which managed the recruitment and enrollment of study subjects and the lifestyle intervention. The lifestyle support center sent a program kit by mail to the eligible subjects in each cluster, inviting them to participate in the study. The kit included an explanation about the study’s aims and protocol, a consent form, and a questionnaire regarding lifestyle and health status. Those who consented to participate and completed the questionnaire were enrolled as study participants at the lifestyle support center, after their eligibility was checked based on their self-reported present and past health conditions and, when available, based on information from physicians in the health care divisions.

### Characteristics of study subjects

As mentioned above, using a questionnaire, subjects in both the intervention and control arms were asked about their lifestyle (diet, exercise habits, and smoking history) and present and past health conditions. They were categorized into following groups by their BMI, based on the WHO Western Pacific Regional Office (WPRO) criteria; <18.5 as “Underweight”, 18.5 to 22.9 as “Normal”, 23.0 to 24.9 as “Overweight”, 25.0 to 29.9 as “Obese I”, and ≥30.0 as “Obese II” [28,29]. To define the Metabolic Syndrome in this study we used the modified criteria of the third report of the National Cholesterol Education Program Expert Panel on Detection, Evaluation, and Treatment of High Cholesterol in Adults (NCEP/ATPIII) [30,31]. When three or more of the following components were present in an individual, the individual was judged to have the Metabolic Syndrome: 1) serum triglycerides ≥150 mg/dL [≥ 1.69 mmol/L];, 2) HDL-cholesterol <40 mg/dL [< 1.04 mmol/L] for men and <50 mg/dL [< 1.29 mmol/L] for women, 3) fasting plasma glucose ≥100 mg/dL [≥ 5.6 mmol/L], 4) blood pressure ≥130/85 mmHg, or use of blood pressure lowering agents, and 5) a BMI of ≥25 kg/m^2^[32]. In 2006, when the baseline data were obtained, waist size was not measured in the majority of the health checkup sites. Therefore, BMI was substituted for waist circumference.

### Goals for lifestyle changes

The goals for lifestyle change are set for each subject from the following four points; 1) habitual exercise (10,000 steps or more per day or 60 min or more per week of accumulated moderate levels of exercise), 2) achievement and maintenance of an appropriate body weight (a 5% reduction in body weight in subjects with a BMI of ≥25 kg/m^2^ or a 3% reduction in subjects with a BMI of 23.0-24.9 kg/m^2^), 3) habitual intake of dietary fiber (five or more dishes of vegetables per day or 350 g or more of vegetables per day ), and 4) restrictions on alcohol intake (1 “go” (180 ml) or less per day in terms of Japanese sake. 1 “go” of Japanese sake contains 23 g of ethanol [33]).

### Lifestyle intervention

**Figure 1:**
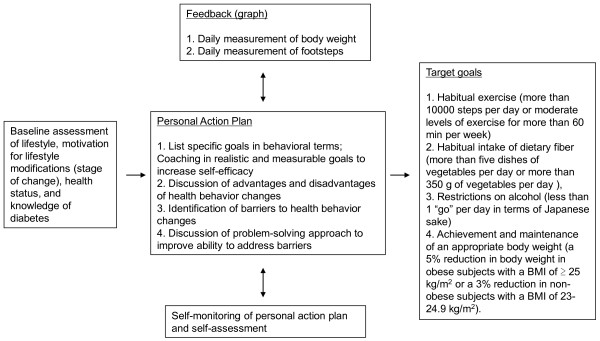
Telephone-delivered lifestyle modification support model: theory matrix.

After setting goals, the intervention and control arms will receive different treatments. For subjects in the control arm, a weight scale (HBF-354 IT-2; Omron Healthcare Co., Ltd., Japan) and a pedometer (HJ-710 IT; Omron Healthcare Co., Ltd., Japan) with a storage function are provided. They will periodically receive newsletters from the lifestyle support center, which run health-related information and messages to encourage them to undergo a health checkup regularly. These are done to minimize the potential for greater attrition from subjects in the control arm. For the subjects in the intervention arm, in addition to the services provided to the control arm, telephone-delivered lifestyle support will be provided over a one-year period through one of the three lifestyle support centers. In addition to phone calls, written information delivered by mail is also used. Subjects monitor achievement of their own personal action plan. They are encouraged to measure body weight and the number of footsteps every day and send the accumulated data to the lifestyle support center monthly via a transmitter (DC-100; JMS Co., Ltd., Japan). The staff will monitor the achievement of subject’s goals regularly and give advice by phone or mail (Figure 1). As mentioned before the intervention is outsourced to private companies. Because the sample size is large, we use three companies. The National Education Association, INC. VISIT HEALTH Co., Ltd., Ltd., Meiji Yasuda System Technology Co., Ltd., and Tokio Marine & Nichido Medical Service Co., Japan will manage 16, 18 and 9 groups, respectively. All study subjects in each group will be managed by the same company. We do not standardize the intervention program. Each company uses its own intervention schedule approved by the study group (Table 1). The intervention is standardized within each company. Public health nurses and registered dieticians employed by the lifestyle support centers have college degree and at least 5 years work experience of the intervention. In addition, we will hold educational sessions on diabetes and its prevention for them and training sessions to improve their skills of telephone counseling with motivational interviewing. As shown in the Table 1, there are considerable differences in the quantity of services among the companies. Participants will receive phone calls at least 3 times, and at most 10 times, over one year with the length of each call being between 15-30 minutes.

**Table 1:** Schedules of telephone counseling of the three lifestyle support centers

	**National Education Association INC. VISIT HEALTH**	**Meiji Yasuda System Technology**	**Tokio Marine & Nichido Medical Service**
Introduction and welcome call	In Week 1	In Month 2	In Month 3
Support calls	In Months 2, 3, 4, 7, and 10.	In Months 3, 4, 5, 6, 7, 8, 9, 10, and 11.	In Months 7 and 12
Advice sheets by mail	No	Monthly, during Month 2-12	Monthly, during Month 2-12
Feedback by graph (body weight and footsteps)	Monthly	Monthly	Monthly
The number of groups in the control/intervention arm	8/8	8/7	4/5
The number of subjects in the control	595/722	413/484	328/298

Data are n.

### Follow-up and outcome

Participants will be followed up over a three- year period using data from an annual health checkup and a questionnaire regarding health and lifestyle. The questionnaires are mailed out to the participants from the lifestyle support center with self-addressed envelopes. If a completed questionnaire is not sent back to the lifestyle support center within two weeks, the lifestyle support center will contact the participant first by mail and then by telephone. We made a manual for this process. The primary outcome is the development of diabetes in participants whose FPG concentration is 100-125 mg/dL (5.6-6.9 mmol/L) at baseline. The development of diabetes is defined as; #1) a rise in FPG to a level equal to or greater than 126 mg/dL (7.0 mmol/L) as revealed in the follow-up annual health checkup, and #2) a diagnosis of diabetes or use of anti-diabetic drugs as reported in the annual questionnaire with confirmation by referring to medical records. Other outcomes are changes in body weight, BMI, plasma glucose, blood pressure, serum lipids, HbA1c, the percentage of subjects with the Metabolic Syndrome, lifestyle, and the development of cardiovascular diseases.

### Dropout and discontinuance

Dropout cases in the present study include; 1) participants who have not undergone an annual health checkup after enrollment, and 2) participants who have lost contact with the study team. Discontinuous cases are defined as; 1) participants who have developed adverse events that make continuance impossible, 2) participants who request to discontinue, 3) participants who are judged inappropriate for continuing the study by the project leader for various reasons.

### Data management

Data management is outsourced to CIMIC Co., Ltd., Japan, a contract research organization offering clinical research management services. All data obtained in the study will be stored in de-identified forms in the data management center and used in conformity with the study aims only. The project leader (HK) has overall responsibility for management of the study data.

### Blinding

Study participants and the staff members are not blinded to the study arm status. Analysts who perform final data analysis will be blinded.

### Sample size

The present study is likely to observe a significantly longer diabetes-free period in the intervention than in the control arm. Thus, the null hypothesis is that the diabetes-free period in the intervention arm is the same as that in the control arm. The sample size(S) needed is calculated using the formula [34,35]; S = (1 + [cluster size − 1] × ICC) × N, where N represents the sample size required when study subjects are randomized individually, and ICC represents an intra-cluster coefficient [36]. Based on the available prospective data from Japanese population the yearly incidence of diabetes among high-risk group varies between 2 and 7% [37,38]. When calculated on the assumption that the annual incidence of diabetes is 4% in the control arm and the intervention reduces the incidence by 50%, N will be 1100 with an alpha of 5% and a power of 90% according to Shoenfeld & Richter [34]. When the ICC and the cluster size (number of individuals in each cluster) are assumed to be 0.02 and 60, S and the number of clusters will be 2398 and 40, respectively. Assuming that the dropout rate is 30%, 3426 subjects are needed. On the assumption that 1) the prevalence of high-risk individuals in each cluster is 10%, and 2) 30% of eligible subjects consent to participate in the study, the total number of health checkup examinees required would be approximately 114,200, and the number of health checkup examinees in each cluster will be approximately 2900. For descriptive analyses of the diabetes-free duration, the Kaplan-Meier method is used.

### Statistical analyses

The analyses are done using Statistical Package for Social Science software version 19.0 (SPSS Inc., Chicago, IL, USA) or SAS version 9.3 (SAS Inc., Cary, NC, USA). The analysis will be done on an intention to treat basis. Survival curves for the development of diabetes will be estimated by the Kaplan–Meier method. The log rank test will be also conducted. We will take into account the clustering effect in the main outcome analysis and sub-analysis using the LWA model (Lee, Wei and Amato) [39-41]. Cox regression analysis will be used to calculate the unadjusted and adjusted HRs and 95% CIs for arm and risk factors. In multivariable Cox analysis, all significant variables selected for the univariate analysis will be used with the criterion of p<0.1. Student's t-test (or Mann-Whitney U-test according to the frequency distribution of the variable) will be used to compare the means (or the distribution) of the two study arms for continuous variables. Chi-square test or chi-square for trend will be used to compare proportions for categorical variables. We do not adjust for the clustering effect for analysis of the secondary outcome. Those cases with missing data will be simply omitted in the relevant analysis. A p value less than 0.05 is considered significant.

## Results

**Figure 2:**
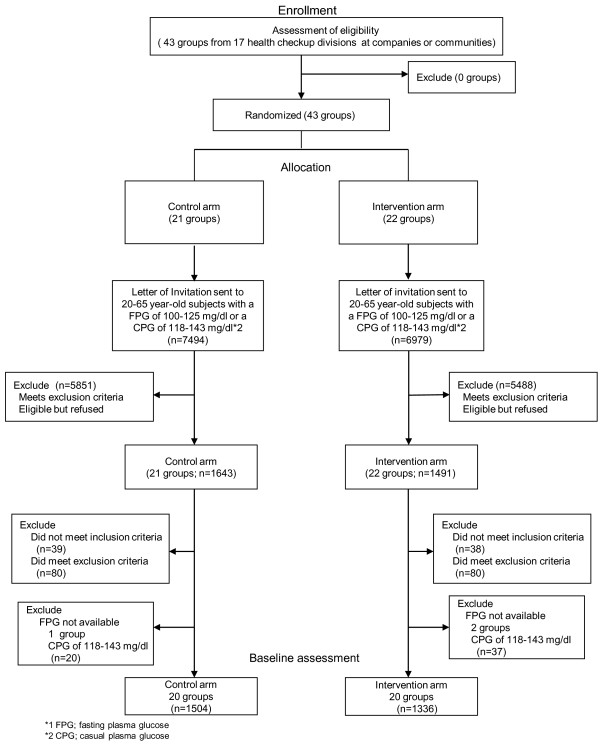
Flowchart of participant recruitment and trial design: main scheme (n=2840).

Forty-three groups, formed from 17 health care divisions at companies or communities across the country, were randomly assigned to a control arm (21 groups) or an intervention arm (22 groups) between March 2007 and February 2008. Figure 2 shows the flow of recruitment of study subjects through annual health checkups. Approximately 230, 000 individuals (male 85%) underwent health checkups by those 43 groups in 2006. Among them, 14,473 subjects (7494 in the control and 6979 in the intervention arm) met the inclusion criteria and received an invitation letter to participate in the study. As a result 1643 subjects from the control and 1491 subjects from the intervention arm consented to participate. Finally, 2897 subjects were enrolled, with 1524 in the control and 1336 in the intervention arm. The overall consent rate of the study was approximately 20% with no difference between the study arms. Among the 2897 subjects, 57 subjects (20 in the control and 37 in the intervention arm) were enrolled with CPG of 118-143 mg/dL 6.6-7.9 mmol/L). As shown in Figure 2, those subjects are not included for the main outcome analysis. The remaining 1504 in the control and 1336 in the intervention arm will be followed up for the development of diabetes (primary outcome). The median (interquartile range) of the group size in the control arm before and after screening for eligibility was 301 (200-442) and 61 (35-88), respectively and those in the intervention arm was 313 (158-587) and 60 (41-94), respectively. There is no difference in group size between arms. In one group in the control and two groups in the intervention arm no participants were enrolled with FPG. Those three groups were not included in the calculation of cluster size. The number of company settings, community settings, and mixed settings in the intervention arm were 16, 3, and 1, respectively. The number of company settings, community settings, and mixed settings in the control arm were 15, 3, and 2, respectively. There were no differences between the arms in the characteristic of the participants in terms of age, sex ratio, FPG levels, BMI, and the prevalence of obesity (Table 2). No difference was found in the prevalence of the Metabolic Syndrome, either (Table 3). All follow-up data will be collected by winter 2012.

**Table 2:** Participant characteristics by randomized intervention assignment

**Variables**	**Control arm**	**Intervention arm**
	(n=1504)	(n=1336)
Age, years	49 (44–54)	49 (44 – 55)
Male,%	85.0	83.8
Body mass index, kg/m^2^	24.0 (22.3 – 25.8)	24.2 (22.3 – 26.3)
WRPO criteria*		
Underweight (less than 18.5 BMI),%	2.1	1.8
Normal (18.5-22.9 BMI),%	33.2	31.1
Overweight (23.0-24.9 BMI),%	28.4	28.1
Obesity I (25.0-29.9 BMI),%	32.0	33.7
Obesity II (Over 30.0 BMI),%	4.3	5.4
Systolic blood pressure, mmHg	125 (114 – 136)	125 (116 – 135)
Diastolic blood pressure, mmHg	80 (71 – 87)	79 (72 – 87)
Total cholesterol, mmol/l	5.4 (4.9 – 6.0)	5.5 (4.8 – 6.1)
HDL-cholesterol, mmol/l	1.5 (1.3 – 1.8)	1.5 (1.2 – 1.8)
Triglyceride, mmol/l	1.3 (0.9 – 1.9)	1.3 (0.9 – 1.8

Values are median (interquartile range or percentage. * The subjects were categorized into following groups based on the WHO West Pacific Regional Office (WPRO) criteria; less than 18.5 BMI, as “Underweight”, 18.5 to 22.9 as “Normal”, 23.0 to 24.9 as “Overweight”, 25.0 to 29.9 as “Obese I” and over 30.0 BMI as “Obese II”.

**Table 3:** Components of metabolic syndrome by randomized intervention assignment and sex

**Variables**	**Control arm**				**Intervention arm**			
	**Men**		**Women**		**Men**		**Women**	
	**(n=1279**		**(n=225)**		**(n=1119)**		**(n=217)**	
1. BMI ≥ 25 kg/m^2^	479	37.5%	67	29.8%	456	40.8%	66	30.4%
2. Hypertension	580	45.3%	75	33.3%	514	45.9%	66	30.4%
3. TG ≥ 150 mg/dl	429	33.5%	30	13.3%	360	32.2%	29	13.4%
4. HDL<40 mg/dl in men, <50 mg/dl in women	77	6.0%	27	12.0%	76	6.8%	24	11.1%
5. Hyperglycemia	1279	100.0%	225	100.0%	1119	100.0%	217	100.0%
Risk factors of metabolic syndrome								
1 factor	365	28.5%	99	44.0%	299	26.7%	97	44.7%
2 factors	416	32.5%	71	31.6%	359	32.1%	66	30.4%
≥3 factors	496	38.8%	55	24.4%	459	41.0%	53	24.4%

Data are number or percentage. Five subjects were excluded from the analyses because of missing data except for fasting plasma glucose.

## Discussion

We have launched this J-DOIT1 trial to test whether goal-focused lifestyle support delivered by healthcare providers via the telephone is feasible and effective for preventing or delaying the development of T2DM in high-risk individuals. Statutory health checkup programs, provided annually by public medical care insurance, would offer significant advantages for carrying out this study. Thus, for the recruitment of study subjects and their follow-up, biochemical and anthropometric data are all obtained from health checkup sites.

### Cluster randomization

In recently reported lifestyle intervention studies, both individual randomization and cluster randomization [42,43] have been used. The cluster randomization design has the advantages of administrative convenience, ease of obtaining the cooperation of investigators, enhancement of subject compliance, and avoidance of treatment contamination. Since study subjects in this trial are employees of the same workplaces or inhabitants of the same communities, we chose cluster randomization to avoid diluting the effect of the intervention. The contamination could occur with individual randomization e.g. by control subjects receiving part of the intervention in a shared environment. Generally, cluster randomized trials, are susceptible to a range of methodological problems including selection bias [18]. Selection bias can be avoided by recruiting and enrolling the study subjects into the study before the groups are allocated to the study arms [44]. In our study design, however, the study subjects were recruited after the clusters were randomly allocated to the intervention or control arm. The reason for nor recruiting and enrolling subjects before randomization was that it was not practical due to the nature of the intervention, in which it takes too long to recruit individuals first. The individuals or the recruiters were not blinded to the allocation status. Careful attention should be paid to the likelihood of selection bias in our sample based on the cluster sizes between the two arms and comparison of the participants.

### Telephone-delivered interventions

Structured intensive lifestyle modification can prevent T2DM in hospital and clinic settings [45-47], and primary healthcare settings [48]. To target young and middle-aged people, who are busy with work, this study employs a non face-to-face intervention using the telephone. Telephone-delivered intervention has a greater accessibility and potential availability of participants for the interview than face-to-face provided support. They facilitate, in a cost-effective manner [37], repeated contact and support for the participant necessary to promote maintenance of physical activity and diet. Thus telephone counseling would make it possible to deliver lifestyle intervention widely, at a low cost, but in a personalized way. There has been increasing interest in lifestyle support using the telephone [49-53]. However, it is unknown whether telephone-delivered support for lifestyle modification by healthcare providers is a feasible and effective way to prevent or delay the development of T2DM. If it is proved effective, lifestyle coaching by healthcare providers using telephone would be a promising tool for reducing the incidence of diabetes.

### Retention

The final sample size (2840 participants) would provide >80% power to detect a 50% reduction in the rate of development of T2DM among participants assigned to the lifestyle intervention with a 5% level of significance (two-sided), after no adjustment for losses in follow-up. The follow-up of participants is scheduled to finish in March 2012. Retention of participating health checkup facilities and subjects are critical for the success of this study. Drop-out rates are generally high in lifestyle programs conducted in primary healthcare clinical settings. To secure enough samples for analysis, participants are encouraged to attend an annual health checkup through a letter from the lifestyle support center. The lifestyle support center gives safety advice to prevent sport injuries which could lead to dropping out of the study.

### BMI and the Metabolic Syndrome

We included not only overweight and obese subjects, but also subjects with a BMI of < 23 kg/m^2^. Therefore, the BMI ranged widely from <18.5 to >30 in our study subjects with an average value of 24.3. Only 39.0% of men and 30.1% of women had a BMI of ≥25 kg/m^2^. Compared with western populations, obesity is less common in our general population [54]. It has also been reported that about 25% of subjects with impaired glucose tolerance have normal or even underweight categories of BMI [36]. It seems that the relationship between BMI and the risk of diabetes is not so straightforward in our population. Thus, we did not set eligibility criteria in terms of BMI. It would be of interest to study the incidence of diabetes and see what strategies are effective to prevent the development of diabetes in those with a lower BMI. In 2008, the concept of the Metabolic Syndrome was introduced in the health checkup program in our country [55]. Mukai et al. suggested that the Metabolic Syndrome significantly increased the risk of incident T2DM, irrespective of the presence or absence of impaired fasting glucose(IFG), and is therefore a valuable tool to identify individuals at high risk of T2DM in the general population in Japan [56]. In this study, we found 39.8% of men and 24.8% of women have ≥3 risk factors for cardiovascular diseases, suggesting they have the Metabolic Syndrome. The present study would allow us to compare the incidence of T2DM in IFG subjects with or without the Metabolic Syndrome in a subanalysis.

### Limitations

This study has several potential limitations. One is that we identified high risk subjects using fasting plasma glucose. We will follow them as to the development of diabetes using fasting plasma glucose determined at annual health checkups and a questionnaire. We do not add any other biochemical examinations such as the oral glucose tolerance test. Therefore, we may miss diabetic subjects having normal fasting but elevated 2 h plasma glucose levels [57-59]. We may also miss subjects with IGT, IFG and IGT, both associated with a substantially increased risk of developing diabetes, are considered to be of a different entity. In the majority of populations thus far studied, IGT is more prevalent than IFG. Thus, we must be careful in interpreting results. It is possible that the efficiency of identifying high-risk subjects will be increased by combining FPG and HbA1c data [60]. This study used results obtained in 2006 annual health checkups as baseline data. At that time, only 58.5% of participating checkup sites included the measurement of HbA1c as a health checkup item. Second, the present study lacks information on the use of drugs, such as fibrate, nicotinic acid, and fish oil, which affect the metabolism of HDL-cholesterol and triglycerides. This may have led us to underestimate the prevalence of the Metabolic Syndrome. Third, participants were predominantly from workplaces. We did not succeed in recruiting more participants from communities. Since men outnumber women in many workplaces in Japan, the study population was predominantly male. This bias may limit the generalizability of our results.

## Conclusions

We have launched J-DOIT1, a nation-wide cluster randomized controlled trial to prevent development of T2DM in high-risk individuals using telephone-delivered intervention. Using annual health checkup data, a large cohort has been developed and successfully randomized. This trial is expected to contribute to evidence-based real-world preventive practices.

## Competing interests

The authors declare that they have no competing interests.

## Authors’ contributions

HK, the project leader, is involved in all aspects of the study. KI, MK, NI, and MN designed the study, and prepared the protocol. NS, KK, YS, KT, and KO were involved in drafting the manuscript. KT, JS, SS and SM participated in statistical analysis. All authors have read and approved the final version of the manuscript.

## Pre-publication history

The pre-publication history for this paper can be accessed here:

http://www.biomedcentral.com/1471-2458/13/81/prepub
